# Pharmacokinetics, Clinical Efficacy, and Safety of Once-Weekly Insulin Icodec: A Structured Narrative Review (2022-2026)

**DOI:** 10.7759/cureus.113509

**Published:** 2026-07-28

**Authors:** Yash Patel, Riddhi Patel, Rutvik Raval

**Affiliations:** 1 Medicine, Smt. Nathiba Hargovandas Lakhmichand (NHL) Municipal Medical College, Ahmedabad, IND; 2 Medicine, Gujarat Cancer Society (GCS) Medical College, Hospital and Research Centre, Ahmedabad, IND; 3 Internal Medicine, Sinai Grace Hospital, Detroit, USA

**Keywords:** continuous glucose monitoring, glycemic control, hypoglycemia, insulin icodec, medication adherence, once-weekly basal insulin, pharmacokinetics, type 2 diabetes mellitus

## Abstract

Suboptimal adherence to daily basal insulin remains a significant barrier to achieving glycemic targets in patients with diabetes mellitus. Insulin icodec is a novel, once-weekly basal insulin analog designed to reduce injection burden and improve treatment compliance. This review evaluates the pharmacological profile, clinical efficacy, and safety of insulin icodec by synthesizing data from the comprehensive Phase 3 ONWARDS clinical trial program. A structured narrative review of recent literature (2022-2026) was conducted, focusing on randomized controlled trials (RCTs), pharmacokinetic studies, and meta-analyses comparing insulin icodec with daily basal insulins (glargine U-100 and degludec). Insulin icodec achieves a half-life of approximately 196 hours through reversible binding to albumin, enabling a once-weekly dosing interval. In patients with type 2 diabetes (T2D), the ONWARDS 1-5 trials demonstrated that icodec provides non-inferior, and in several cohorts, superior, reductions in glycated hemoglobin (HbA1c) compared to daily basal insulins, with a comparable risk of clinically significant or severe hypoglycemia. Time-in-range (TIR) metrics were significantly improved. However, in patients with type 1 diabetes (T1D) (ONWARDS 6), while glycemic efficacy was non-inferior, icodec was associated with a statistically significant increase in the risk of severe or clinically significant hypoglycemia compared to insulin degludec. Insulin icodec may represent an important advancement in the management of T2D, offering improved glycemic control with a substantially reduced injection burden. While its use in T1D requires cautious consideration due to hypoglycemia risks, icodec has the potential to become a preferred standard of care for insulin-requiring T2D globally.

## Introduction and background

Exogenous insulin therapy has long been the cornerstone for managing both Type 1 and Type 2 diabetes, essential for maintaining glycemic control and preventing acute hyperglycemic complications. Modern therapeutic standards have evolved significantly, moving from short-acting formulations to refined recombinant insulin analogs such as neutral protamine hagedorn (NPH), and subsequently to ultra-long-acting basal insulins like glargine and degludec. These advancements have markedly improved the stability of insulin delivery, mimicking physiological basal insulin secretion more precisely and greatly reducing the frequency of hypoglycemia compared to earlier iterations.

However, despite these pharmacological innovations, a vast number of patients still struggle to reach therapeutic glycated hemoglobin (HbA1c) targets. A substantial barrier remains in the form of "clinical inertia," a phenomenon largely driven by the psychological and physical strain of daily self-administration. Factors such as injection site fatigue, needle phobia, forgetfulness, and the perceived social and physical burden of constant daily management frequently result in suboptimal treatment adherence. This lack of compliance directly corresponds to poor long-term glycemic outcomes, enhanced risks of microvascular complications, and higher healthcare use [1].

To overcome these persistent barriers to adherence, the development of once-weekly basal insulins may represent a pivotal evolution in diabetes care [2]. Insulin icodec has emerged as the first-in-class, once-weekly basal insulin analog engineered to directly address the limitations of daily basal regimens [3]. By using a unique molecular structure, specifically a C20 fatty diacid moiety that promotes strong, reversible albumin binding, icodec achieves an extended half-life of approximately 196 hours [4,5]. This innovation fundamentally alters the treatment landscape by reducing the basal injection frequency from 365 to 52 injections per year. By simplifying the regimen, icodec aims to improve patient compliance, enhance overall quality of life, and maintain consistent glycemic control, thereby mitigating the compliance gaps that have historically plagued daily basal insulin therapy [6].

The clinical significance of insulin icodec extends beyond mere convenience; it signals an important advancement in how clinicians and patients approach the long-term management of diabetes. While the reduction in injection burden is undoubtedly a major advantage for patients with T2D, the clinical adoption of icodec requires a sophisticated understanding of its pharmacokinetic profile. The shift toward a weekly dosing interval offers a robust solution for patient engagement, yet it necessitates careful titration and an awareness of the risks, particularly concerning the increased hypoglycemic events observed in specific populations like those with T1D [7]. Moving forward, the successful integration of icodec into standard care will rely not just on its ability to lower HbA1c, but on the development of rigorous patient selection and metabolic monitoring protocols. This paper reviews the molecular design, pharmacokinetics, and comprehensive Phase 3 clinical data surrounding insulin icodec, contextualizing its role in the future of modern diabetes care.

## Review

Methods and literature search strategy

A systematic exploration of the available literature was performed to assess the pharmacological characteristics, safety, and clinical efficacy of once-weekly insulin icodec. To adhere to the specific scope of this analysis, this manuscript was structured as a structured narrative review. Therefore, Preferred Reporting Items for Systematic Reviews and Meta-Analyses (PRISMA) frameworks were not applied; instead, a targeted qualitative synthesis of the highest-level evidence was conducted.

A narrative review was conducted in accordance with the Scale for the Assessment of Narrative Review Articles (SANRA) criteria [8]. A comprehensive literature search was conducted across primary biomedical repositories, including PubMed/MEDLINE, Embase, the Cochrane Central Register of Controlled Trials (CENTRAL), and ClinicalTrials.gov [9-14]. This search spanned the period from January 2022 to April 2026, ensuring the inclusion of the most contemporary Phase 3 evidence and post-marketing findings. The identification of studies relied on a curated set of Medical Subject Headings (MeSH) and descriptive terms: ("insulin icodec" OR "once-weekly basal insulin" OR "icodec") paired with ("Type 2 Diabetes" OR "Type 1 Diabetes" OR "ONWARDS" OR "pharmacokinetics" OR "hypoglycemia"). To ensure comprehensiveness, reference lists from identified systematic reviews and meta-analyses were scrutinized manually for supplemental evidence.

Candidate studies were rigorously vetted using the Population, Intervention, Comparison, and Outcome (PICO) framework. The population included adults (aged 18 or older) diagnosed with T1D or T2D who required basal insulin intervention, including both those initiating therapy and those already on insulin. The intervention was the subcutaneous delivery of insulin icodec on a once-weekly schedule, compared against standard once-daily basal analogs (primarily insulin glargine and insulin degludec). The change in HbA1c served as the primary measure of efficacy, alongside continuous glucose monitoring (CGM) parameters (notably time-in-range (TIR)) and hypoglycemia incidence. Research involving animal models, early-phase dose-finding studies (except for those clarifying unique pharmacokinetic mechanisms), unvetted pre-prints, and trials of alternative once-weekly products like efsitora alfa were omitted.

Two investigators independently performed initial title and abstract screening, subsequent full-text evaluation, and data extraction from the included studies. Disparities regarding study inclusion or data synthesis were settled through collaborative consensus with the designated senior author. Articles meeting the inclusion criteria were precisely reviewed for data on pharmacokinetic properties (half-life and albumin-binding affinity), primary glycemic efficacy (HbA1c reduction and TIR metrics), safety profiles (incidence of severe and clinically significant hypoglycemia), and transitioning protocols. A total of 27 peer-reviewed publications, regulatory reports, and methodological guidelines were included in the final synthesis, comprising six Phase 3 randomized controlled trials from the ONWARDS clinical program alongside their six corresponding trial registries, three meta-analyses, two molecular and pharmacokinetic evaluations, one real-world cohort study, seven comprehensive clinical reviews, and two methodological assessment scales.

Molecular design and pharmacokinetics

Amino Acid Substitutions and Structural Stability 

Insulin icodec is a novel insulin analog, specifically designed to provide a steady, prolonged glucose-lowering effect with minimal peak-to-trough variations over a full 168-hour weekly dosing interval [4]. Scientists engineered fundamental amino acid substitutions into human insulin to achieve the ultra-long pharmacokinetic profile of insulin icodec. Its highly specialized structural modifications include three specific amino acid substitutions (A14E, B16H, B25H) and the strategic deletion of the B30 threonine amino acid (desB30).

These precise alterations in sequence provide a dual biochemical purpose. First, the substitutions at B16 and B25 purposely alleviate the molecule's binding affinity for the insulin receptor. While appearing paradoxical, this decreased affinity is a critical design feature; it deliberately slows receptor-mediated internalization and intracellular degradation, thereby greatly decreasing the physiological clearance rate of the analog. Insulin icodec is provided at a higher molar concentration to compensate for the lower receptor affinity and maintain therapeutic efficacy. Second, the A14E substitution and the deletion of the B30 amino acid conjointly enhance the general molecular stability of the analog, making it highly resistant to enzymatic degradation by prevalent circulating and interstitial proteases. These modifications assure the structural integrity of the insulin molecule during its prolonged presence in the systemic circulation [5].

Acylation and the Albumin-Binding Depot 

Targeted acylation of the insulin molecule is the primary mechanism granting insulin icodec its ultra-long duration of action. This involves the covalent attachment of a large, lipophilic C20 icosanedioic fatty diacid side chain to the lysine residue at position B29 [6]. This stands for a significant biochemical transformation from earlier-generation basal insulins; for context, insulin detemir implements a C14 fatty acid, and insulin degludec implements a C16 fatty diacid. The larger C20 moiety in icodec promotes a considerably stronger binding affinity to albumin.

Essentially, this fatty diacid is attached to the insulin backbone via a highly specific hydrophilic spacer consisting of γ-glutamic acid and two OEG (oligoethylene glycol) motifs. This spacer acts as a flexible hinge, preventing steric hindrance and ensuring the molecule retains its ability to interact with the insulin receptor once freed from albumin. This acylation promotes strong, yet highly reversible, non-covalent binding to both interstitial albumin at the injection site and circulating albumin in the plasma following subcutaneous injection. This interaction makes sure that more than 99% of the administered insulin icodec remains in a pharmacologically inert, albumin-bound state, establishing a massive, slow-release vascular reservoir.

Absorption, Distribution, and Clearance Kinetics 

The absorption kinetics of insulin icodec are peculiarly subjected to this albumin-binding mechanism rather than the delayed subcutaneous precipitation or multi-hexamer formation seen in glargine and degludec, respectively. The therapeutic effect of insulin icodec is driven by the gradual, continuous release of active, unbound monomers from the albumin carrier, which diffuse down their concentration gradient into the systemic circulation.

Insulin icodec attains an extended elimination half-life of approximately 196 hours (8.1 days) and displays a low apparent volume of distribution closely matching the plasma volume (approximately 0.14 L/kg) because of this profound depot effect and the engineered reduction in receptor-mediated clearance [5].

Steady-state plasma concentrations are typically achieved after three to four weekly injections because of this pharmacokinetic profile. Once steady-state is reached, the pharmacokinetic and pharmacodynamic profiles demonstrate an incredibly even distribution of glucose-lowering activity across the entire seven-day dosing interval. Clamp studies demonstrate that icodec effectively diminishes the peak-to-trough fluctuations and late-week "wear-off" effects historically noticed with once-daily basal insulins, providing a continuous therapeutic effect that promotes smoother, more reliable glycemic control throughout the week [3,6].

Clinical efficacy: The ONWARDS trial program

The comprehensive ONWARDS (once-weekly basal insulin icodec) Phase 3 clinical trial program rigorously evaluated the safety, clinical efficacy, and therapeutic utility of once-weekly insulin icodec in more than 4,000 participants across six global, randomized, treat-to-target trials. In this global series, a wide variety of patient populations traversing the entire metabolic spectrum of diabetes was included to ensure reliable clinical generalizability across both primary and specialized care settings [15-21].

Type 2 Diabetes Mellitus: Insulin-Naïve Populations 

Addressing clinical inertia requires mediating effectively at the point of insulin initiation, a phase notoriously impeded by patient anxiety regarding daily injections. The ONWARDS 1, 3, and 5 trials specifically focused on insulin-naïve adults with T2D transitioning to injectable therapy.

In the 78-week ONWARDS 1 trial (n=984), a randomized, open-label, treat-to-target study, once-weekly icodec demonstrated statistically superior HbA1c reductions. Patients achieved a mean decrease of 1.55% compared to 1.35% for those on once-daily insulin glargine U-100 at the primary 26-week endpoint, a benefit that was enduringly maintained through week 78 [15]. In addition, icodec achieved superior TIR metrics, a finding confirmed by glycemic analysis using CGM data from this trial. Patients receiving icodec spent significantly more time in their target glycemic zones (70-180 mg/dL) compared to the glargine cohort, without a corresponding increase in time spent in the clinically significant hypoglycemic range (<54 mg/dL), indicating smoother overall metabolic profiles [16].

The 26-week ONWARDS 3 trial (n=588) supported these findings by employing a rigorous double-blind, double-dummy design to assess icodec against once-daily insulin degludec (an ultra-long-acting daily comparator). This trial abolished the psychological bias associated with weekly versus daily administration by using placebo injections to mask the injection frequency. Once again, icodec demonstrated superior HbA1c reduction in insulin-naïve patients, verifying its inherent pharmacological efficacy extends beyond direct comparisons to earlier-generation glargine [17].

Finally, ONWARDS 5 (n=1,085) utilized a practical, real-world trial design over 52 weeks. Unlike highly controlled traditional trials, this study integrated a DoseGuide app, a clinical decision support software incorporated into patients' smartphones that algorithmically guided weekly titration based on patient-logged fasting plasma glucose values. This trial authenticated that icodec achieved superior HbA1c reductions and high patient adherence rates, translating effectively outside of the stringent clinical trial setting into standard, real-world ambulatory care [18].

Type 2 Diabetes Mellitus: Insulin-Experienced Populations 

For individuals needing a transition from an already established daily basal therapy, ONWARDS 2 and 4 trials evaluated the efficacy and safety of switching to a weekly regimen.

The ONWARDS 2 study (n=526) included T2D participants switching directly from existing daily basal insulin therapy (primarily glargine or degludec) to icodec. The transition to once-weekly insulin icodec demonstrated not only strict non-inferiority, but statistically superior HbA1c reductions after 26 weeks when compared to remaining on once-daily insulin degludec. In addition, structured patient-reported outcome measures, especially the Diabetes Treatment Satisfaction Questionnaire (DTSQ), unveiled substantially higher treatment satisfaction scores among the icodec cohort, emphasizing the psychosocial benefit of decreasing the annual basal injection burden from 365 to 52 [19].

ONWARDS 4 (n=582) explored a substantially more complex regimen, assessing a full basal-bolus strategy utilizing icodec against glargine U-100, both combined with multiple daily injections of mealtime insulin aspart, in patients with advanced T2D. Icodec successfully attained strict non-inferiority in glycemic control, demonstrating that a once-weekly basal backbone can effectively and safely anchor an intensive multiple daily injection (MDI) regimen [20]. Post-hoc analyses of these switch trials further confirmed robust, sustained CGM-based improvements across the board, verifying that the 196-hour half-life of icodec provides sufficient basal coverage to support daily prandial spikes [21].

Type 1 Diabetes Mellitus 

The ONWARDS 6 investigation (n=582) analyzed the use of insulin icodec within an intensive basal-bolus framework specifically for patients with T1D over a 52-week period [7]. While icodec successfully attained non-inferior glycemic efficacy in HbA1c reduction at 26 weeks relative to daily insulin degludec, this clinical benefit came with important safety precautions that highlight the unique pathophysiology of T1D.

Because patients with T1D do not have the endogenous insulin reserves and counter-regulatory mechanisms present in T2D, they need precise, daily titrations of basal insulin to adapt to variations in physical activity, acute illness, or dietary shifts. The fixed, ultra-long-acting albumin-bound depot of icodec lacks this essential pharmacokinetic flexibility. Consequently, while overall HbA1c improved, the use of icodec in T1D was accompanied by a statistically significant increase in the incidence of combined severe (level 3, requiring external assistance for recovery) and clinically significant (level 2, glucose level <54 mg/dL) hypoglycemic events. Icodec's clinical utility is currently optimized for, and largely restricted to, T2D because of this physiological mismatch between drug kinetics and T1D basal requirements.

Safety, tolerability, and organ impairment

Hypoglycemia, Immunogenicity, and General Tolerability in T2D 

The clinical safety profile of insulin icodec across the T2D-centric ONWARDS trials demonstrated that the incidents of clinically significant (Level 2; blood glucose <54 mg/dL) and severe (Level 3; requiring external assistance) hypoglycemia were comparable to daily analogs [22,23]. The gradual-release pharmacokinetic profile of icodec plays an important role in minimizing nocturnal hypoglycemic dips that often complicate basal insulin therapy. While nuanced numerical discrepancies in mild hypoglycemic events were observed during the early titration phases (Weeks 1-4) as the albumin-bound depot reached a steady state, these momentary events were effectively alleviated by optimizing patient-specific dosing algorithms and utilizing more conservative initial conversion ratios [24].

Regarding general tolerability, participants treated with icodec demonstrated modest, expected weight gain. This is consistent with the well-documented anabolic nature of insulin initiation, which promotes lipogenesis, inhibits lipolysis, and decreases the caloric wasting associated with glycosuria. Importantly, despite the notable structural modifications to the insulin molecule and the addition of the large C20 fatty diacid, no significant rise in immunogenicity or the formation of neutralizing anti-insulin antibodies was observed. Moreover, the formulation's high concentration (U-700) facilitated remarkably small injection volumes (e.g., a 70-unit dose requires only 0.1 mL of fluid), which contributed to the near absence of localized injection-site reactions, hypersensitivity, or lipohypertrophy [6,25].

Pharmacokinetics in Renal and Hepatic Impairment 

Patients with long-standing diabetes or advanced age are frequently afflicted with end-organ damage, making the systemic clearance of diabetic medications a principal safety concern. Classically, decreasing renal function leads to a prolonged insulin half-life and an elevated risk of uremic hypoglycemia due to reduced renal insulin degradation. However, specific pharmacokinetic clamp studies have evaluated icodec's safety across special populations with reassuring results.

The ultra-long half-life (t_1/2_), maximum concentration (Cmax), and overall area under the curve (AUC) of icodec remain largely consistent across varying degrees of renal impairment (from mild chronic kidney disease to end-stage renal disease necessitating hemodialysis) and hepatic impairment. This stability is primarily attributed to icodec's massive albumin-bound reservoir, which efficiently buffers against sudden alterations in systemic clearance rates [24]. Consequently, no specific empirical dose adjustments or reductions are required for these often-vulnerable cohorts because of this unique pharmacokinetic resilience. This dramatically simplifies clinical integration and prescribing protocols for patients simultaneously battling metabolic dysfunction-associated steatotic liver disease (MASLD) or diabetic nephropathy.

The Physiological Challenge of Type 1 Diabetes

In contrast to the highly favorable safety profile observed in T2D, the complex interplay between T1D pathophysiology and the inherent pharmacokinetic persistence of a weekly insulin presents severe clinical challenges [7,26]. Individuals with T1D entirely lack endogenous insulin reserves. More crucially, they suffer from malfunctioning paracrine counter-regulatory mechanisms, most notably, the loss of the physiological alpha-cell glucagon response to falling blood glucose, leaving them entirely dependent on exogenous carbohydrate intake and systemic epinephrine to buffer glycemic drops.

The daily, and sometimes hourly, fluctuations in basal requirements typical of T1D are fundamentally incompatible with the fixed albumin-bound reservoir of icodec. Because icodec creates a steady, continuous monomeric release that cannot be immediately truncated, it exhibits pharmacological rigidity. If a patient with T1D engages in unplanned, profound physical exertion (which dramatically enhances skeletal muscle insulin sensitivity) or experiences a gastrointestinal illness with compromised enteral intake, their basal insulin cannot be down-titrated for that day or the next. The continuous release of active insulin from the albumin depot will persistently drive glucose into cells, leaving patients with T1D susceptible to prolonged, refractory hypoglycemic episodes that are difficult to reverse.

Clinical integration: Dosing and transitioning protocols

Formulation and the U-700 Pen Device 

Insulin icodec is formulated at a highly concentrated 700 units/mL (U-700) to facilitate a once-weekly dosing regimen without requiring large injection volumes. This dense formulation ensures that a standard weekly dose requires a minimal subcutaneous fluid volume (e.g., a 70-unit dose equates to merely 0.1 mL of fluid), which substantially minimizes tissue distention, medication leakage, and injection-site pain. Its clinical administration is standardized using a specialized, proprietary pen injector to ensure absolute patient safety and prevent dosing errors. This device dials and displays the dose purely in standard "units," completely abstracting the U-700 concentration away from the patient. Clinically, 1 "unit" of icodec on the pen dial is equipotent to 1 unit of conventional U-100 daily basal analogs, requiring no complex mental math from the prescriber or the patient [3].

Initiation and Titration in Insulin-Naïve Patients 

For insulin-naïve adults with T2D starting injectable therapy, the recommended starting dose is an intuitive 70 units delivered subcutaneously once weekly (which is therapeutically equivalent to a starting dose of 10 units per day of a daily basal insulin). Straightforward weekly dose adjustments are guided by self-monitored fasting plasma glucose (FPG) trends following initiation. A standard titration algorithm typically involves adjusting the weekly icodec dose by an increase or decrease of 20 units based on the average FPG of the prior three days, aiming for a strict clinical target of 80-130 mg/dL.

The "Loading Phase" for Insulin-Experienced Patients 

A specialized, biphasic conversion protocol is pharmacokinetically needed to rapidly introduce the necessary albumin-bound depot when a patient transitions from an established once-daily basal regimen (such as degludec or glargine). If a patient simply takes a 1:1 weekly equivalent of their daily insulin, the slow release of monomers from the albumin pool leads to the generation of sub-therapeutic plasma insulin levels during the first seven to 14 days, leading to rebound hyperglycemia.

A Week 1 "Loading Phase" is used to avoid this. The initial loading dose is calculated by taking 1.5 times the total weekly equivalent of the previous daily basal dosage (e.g., a patient taking 20 units of glargine daily requires 140 units weekly; the Week 1 loading dose is 140×1.5=210 units). This one-time bolus rapidly and safely saturates the circulating albumin pool, constituting immediate steady-state basal coverage. From the second week onward (the Maintenance Phase), the regimen regresses to a standard 1:1 weekly conversion (the previous total daily dose multiplied by seven, which in this example would be 140 units weekly).

Missed Doses and "Sick Day" Management 

The incorporation of a weekly insulin needs a paradigm shift in how clinicians educate patients on sick day rules and missed doses. If a patient accidentally misses their scheduled weekly dose, the protocol directs that they take it as soon as they remember, provided there are at least three days (72 hours) before the next regularly scheduled dose. If less than three days remain, the missed dose should be skipped to prevent depot stacking and severe hypoglycemia.

Crucially, proactive dose reductions or the withholding of basal insulin are strictly dissuaded during short-term transient illnesses (e.g., gastroenteritis or febrile illnesses causing poor oral intake). Skipping or lowering a dose of icodec will not provide any immediate relief from acute hypoglycemia because of the ultra-long 196-hour half-life. The depot cannot be "shut off." Instead, glycemic instability or hypoglycemic trends during these vulnerable periods must be managed reactively and dynamically via supplemental carbohydrate intake, adjustments to short-acting prandial insulins, or the temporary reduction of concomitant oral hypoglycemic agents (such as SGLT2 inhibitors or sulfonylureas) [26].

Clinical implications and the future of diabetes care

Overcoming Clinical Inertia and the Psychological Burden 

The clinical adoption of insulin icodec may represent an important advancement in modern-day diabetes care. The therapeutic obligation of basal insulin has relied on a stringent 365-injection annual regimen for decades. By transitioning this burden to a highly streamlined 52-injection schedule, icodec addresses an important contributor to clinical inertia. This well-documented phenomenon occurs where both physicians and patients delay the necessary initiation of insulin therapy by three to five years due to needle phobia and psychological resistance [2]. This drastic diminution in injection frequency increases treatment satisfaction, enhances patient-reported outcomes, and supports long-term medication adherence by removing the daily physical and cognitive friction of diabetes management.

High-Yield Populations and Healthcare Infrastructure 

While icodec offers lifestyle flexibility for the general T2D population, its clinical implications are most meaningful for specific high-risk cohorts. Icodec is positioned to become a preferred first-line basal intervention for frequent travelers, those experiencing severe needle-related anxiety, or individuals with busy, unpredictable occupational schedules.

Moreover, this once-weekly profile offers extensive systemic benefits for healthcare infrastructure and geriatric patients. For geriatric patients experiencing cognitive decline, visual impairments, or dexterity issues (such as severe osteoarthritis), daily self-administration is often difficult, rendering them entirely dependent on visiting home-health nurses or family caregivers. A once-weekly injection greatly decreases caregiver burden. On a macro-economic scale, transitioning patients to insulin icodec in nursing homes and long-term care facilities has the potential to decrease the daily nursing time required for medication rounds, reduce the accumulation of biohazardous sharps waste across the healthcare system, and decrease the statistical probability of daily dosing errors.

Digital Health Integration and the Evolving Landscape 

The future of diabetes care lies at the intersection of digital health integration and ultra-long-acting pharmacotherapy. The successful implementation of the DoseGuide app in the ONWARDS 5 trial emphasizes how once-weekly insulins can be paired with clinical decision support algorithms to semi-automate the titration process. As CGMs become the standard of care, the integration of real-time CGM data with weekly insulin dosing algorithms will likely enable predictive adjustments to the weekly depot, further alleviating hypoglycemic risks.

The Educational Imperative 

However, an educational undertaking within the medical community is needed to realize this future. Primary care physicians, endocrinologists, and emergency medicine clinicians should unlearn daily insulin mechanics to incorporate the shift from a 24-hour to a 196-hour half-life. Protocols for perioperative management, transitions of care during hospital admissions, and "sick day" rules must be completely rewritten to account for the unyielding albumin-bound depot. As insulin icodec transforms the standard of care, ensuring comprehensive provider education on these distinct pharmacokinetic realities will be a vital factor in guaranteeing patient safety during this therapeutic transition.

## Conclusions

Insulin icodec is a promising once-weekly basal insulin analog that addresses one of the important contributors to clinical inertia by significantly reducing the treatment burden associated with daily injections. By using a sophisticated C20 icosanedioic fatty diacid moiety to promote reversible albumin binding, this novel analog attains a 196-hour pharmacokinetic duration with minimal peak-to-trough variations. The comprehensive ONWARDS Phase 3 clinical trial program has demonstrated that transitioning to a 52-injection annual schedule does not jeopardize metabolic control. Insulin icodec constantly demonstrated non-inferior, and frequently superior, HbA1c reductions and TIR metrics compared to established daily basal analogs in patients with Type 2 diabetes. This was accomplished while maintaining a comparable safety profile regarding severe hypoglycemia and demonstrating pharmacokinetic stability across varying degrees of renal and hepatic impairment. Despite these benefits, its use in Type 1 diabetes is currently restrained by a complex hypoglycemic risk profile; the pharmacological rigidity of a massive albumin-bound depot is inherently incompatible with the dynamic basal requirements and absent counter-regulatory mechanisms characteristic of T1D. Eventually, by offering reliable glycemic efficacy combined with a reduction in treatment burden, insulin icodec represents an important advancement in modern, patient-centric metabolic management for insulin-requiring patients with Type 2 diabetes globally.

## Appendices

Table 1 summarises the core literature, specifically focusing on the ONWARDS Phase III clinical trial program and targeted pharmacokinetic analyses, that informed the clinical efficacy and safety synthesis.  

**Table 1:** Summary of key included studies. T2D=Type 2 Diabetes; T1D=Type 1 Diabetes; RCT=randomized controlled trial; HbA1c=glycated hemoglobin; TIR=Time-in-Range.

Author(s) & citation	Year	Study design	Patient population	Key metabolic and safety findings
Russell-Jones et al. [7]	2023	Phase III RCT (ONWARDS 6)	T1D (basal-bolus strategy)	Non-inferior HbA1c reduction at 26 weeks relative to daily insulin degludec; however, accompanied by a statistically significant increase in the risk of severe or clinically significant hypoglycemia.
Rosenstock et al. [15]	2023	Phase III RCT (ONWARDS 1)	T2D (insulin-naïve)	Superior HbA1c reduction (1.55% vs 1.35%) and Time-in-Range (TIR) metrics vs. once-daily insulin glargine U100 at 78 weeks.
Lingvay et al. [17]	2023	Phase III RCT (ONWARDS 3)	T2D (insulin-naïve)	Superior HbA1c reduction at 26 weeks compared to once-daily insulin degludec.
Bajaj et al. [18]	2023	Phase III RCT (ONWARDS 5)	T2D (insulin-naïve)	Pragmatic, real-world trial utilizing a DoseGuide app; demonstrated superior HbA1c reductions compared to standard daily basal insulins.
Philis-Tsimikas et al. [19]	2023	Phase III RCT (ONWARDS 2)	T2D (insulin-experienced)	Statistically superior HbA1c reductions after 26 weeks vs. once-daily insulin degludec; significantly higher treatment satisfaction scores.
Mathieu et al. [20]	2023	Phase III RCT (ONWARDS 4)	T2D (basal-bolus strategy)	Strict non-inferiority in glycemic control compared to glargine U100, achieved with a substantially reduced basal injection frequency.
Haahr et al. [24]	2024	Pharmacokinetic cohort analysis	Patients with varying degrees of renal or hepatic impairment	Demonstrated that the ultra-long half-life and clearance of icodec remain unaffected by organ impairment; no specific dose adjustments required.

As this is a narrative review, a simplified, domain-based risk of bias assessment (adapted from the Cochrane Risk of Bias tool [27]) was performed for the primary randomized controlled trials evaluated in the clinical synthesis (Table 2).

**Table 2:** Risk of bias assessment for key clinical trials. Note: While some ONWARDS trials (e.g., ONWARDS 6) utilised open-label designs (high risk for performance bias), the primary metabolic outcomes assessed in this review (HbA1c decline and CGM-derived Time-in-Range measured by continuous monitors) are highly objective parameters. Therefore, detection bias for the primary glycemic endpoints was graded as low for all included trials. HbA1c: Glycated hemoglobin; CGM: continuous glucose monitoring.

Study and citation	Random sequence generation (selection bias)	Allocation concealment (selection bias)	Blinding of outcome assessment (detection bias)*	Incomplete outcome data (attrition bias)	Selective reporting (reporting bias)	Overall risk of bias
Russell-Jones et al. [7]	Low	Low	Low	Low	Low	Low
Rosenstock et al. [15]	Low	Low	Low	Low	Low	Low
Lingvay et al. [17]	Low	Low	Low	Low	Low	Low
Bajaj et al. [18]	Low	Low	Low	Low	Low	Low
Philis-Tsimikas et al. [19]	Low	Low	Low	Low	Low	Low
Mathieu et al. [20]	Low	Low	Low	Low	Low	Low
